# Potential role of two novel agonists of thyroid hormone receptor‐β on liver regeneration

**DOI:** 10.1111/cpr.12808

**Published:** 2020-04-29

**Authors:** Andrea Perra, Marta Anna Kowalik, Lavinia Cabras, Massimiliano Runfola, Simona Sestito, Cristina Migliore, Silvia Giordano, Grazia Chiellini, Simona Rapposelli, Amedeo Columbano

**Affiliations:** ^1^ Department of Biomedical Sciences Unit of Oncology and Molecular Pathology University of Cagliari Cagliari Italy; ^2^ Department of Pharmacy University of Pisa Pisa Italy; ^3^ Department of Pathology University of Pisa Pisa Italy; ^4^ Department of Oncology University of Turin Turin Italy; ^5^ Institute‐FPO IRCCS Italy

**Keywords:** hepatocyte proliferation, regenerative medicine, thyromimetics, TRβ selective agonists

## Abstract

**Objectives:**

Although the hepatomitogenic activity of triiodothyronine (T3) is well established, the wide range of harmful effects exerted by this hormone precludes its use in liver regenerative therapy. Selective agonists of the beta isoform of thyroid hormone receptor (TRβ) do not exhibit T3‐induced cardiotoxicity and show a good safety profile in patients with NASH. The aim of this study was to investigate whether two novel TRβ agonists, the prodrug TG68 and the active compound IS25 could stimulate hepatocyte proliferation without T3/TRα‐dependent side effects.

**Methods:**

Rats were treated with three different doses (12.5, 25 and 50 μg/100 g body weight) for one week. Hepatocyte proliferation, liver injury and serum biochemical parameters were measured by immunohistochemistry, qRT‐PCR and Western blot.

**Results:**

Both drugs increased hepatocyte proliferation as assessed by bromodeoxyuridine incorporation (from 14% to 28% vs 5% of controls) and mitotic activity. Enhanced proliferation occurred in the absence of significant signs of liver injury as shown by lack of increased serum transaminase levels or of apoptosis. No cardiac or renal hypertrophy typically associated with treatment with T3 was observed. Importantly, no proliferation of pancreatic acinar cells, such as that seen after administration of T3 or the TRβ agonist GC1 was detected following either TG68 or IS25, demonstrating the hepato‐specificity of these novel TRβ agonists.

**Conclusions:**

The present study shows that TG68 and IS25 induce massive hepatocyte proliferation without overt toxicity. Hence, these agents may have a significant clinical application for regenerative therapies in liver transplantation or other surgical settings.

## 1 INTRODUCTION

**Figure 1:**
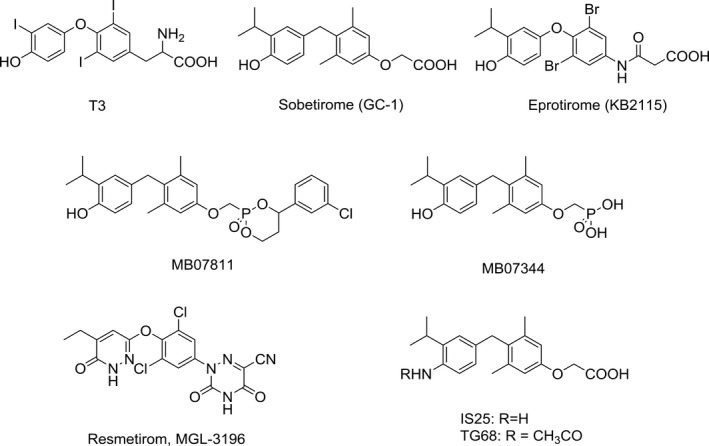
Structures of T3 and novel thyroid hormone receptor β agonists

The liver plays a fundamental role in the metabolic functions of the body and possesses a remarkable capacity to adapt to metabolic perturbations. Liver regeneration after partial hepatectomy (PH) typifies one such capacity in that the organ shifts instantly from a quiescent state to a rapidly growing one.^1^ The unique ability of the liver to rapidly restore its mass has long been a focus of research not only for scientific purposes but also for clinical applications. Indeed, it has been shown to be lifesaving in cases of end‐stage liver disease and in the transplantation of partial liver grafts.^2^ However, the capacity of liver to regain its original mass and function is limited, making the requirement for a sufficient liver volume a factor limiting the application of liver surgery. Therefore, search for agents capable of inducing hepatocyte proliferation in the absence of liver injury and able to accelerate the replacement of cell loss has been actively pursued.^3^ Among the mitogenic agents so far identified, ligands of nuclear receptors of the steroid/thyroid hormone superfamily are the most represented and include agonists of peroxisome proliferator activated receptor‐α (PPARα), constitutive androstane receptor (CAR), *all‐trans* retinoic acid receptors (RARs) and thyroid hormone receptors (TRs).^3^, ^4^, ^5^ Since T3 is a naturally occurring mitogen,^5^, ^6^, ^7^ it has been the focus of great interest as a potential therapeutic agent. Unfortunately, its use may cause significant side effects, in particular cardiac dysfunction, that is tachycardia, arrhythmias and precipitation of ischaemic episodes or heart failure and thyrotoxicosis.^8^ These and other adverse effects have strongly limited the possible use of T3 as a therapeutic agent. Since it is believed that most of the adverse effects of T3 are mediated by the α isoform of TR,^9^, ^10^ several efforts have been made to develop T3 analogues that could produce a selective modulation of the β isoform (TRβ), therefore allowing to circumvent many of these undesirable events while producing beneficial effects, including triglyceride, cholesterol, obesity and body mass lowering. Among the several TRβ‐selective T3 analogues so far generated, GC‐1, KB2115 and the Hep‐Direct prodrug MB07811^11^, ^12^, ^13^ have reproduced most of the beneficial effects of T3, in the absence of deleterious effects (Figure 1). Experimental studies showed that treatment with GC‐1 causes a reduction in triglyceride levels greater than that produced by equimolar doses of T3.^14^ These effects were achieved at doses devoid of relevant side effects on heart rate and that did not cause muscle loss or an increase in the overall catabolic state.^15^

Indeed, GC‐1 has been shown to prevent the development and progression of rat fatty liver by increasing mitochondrial and peroxisomal fatty acid β‐oxidation and reduced levels of inflammatory markers.^16^ Similarly, MB07344 and its prodrug MB07811 showed anti‐steatotic activity in different animal models.^17^, ^18^ Due to their beneficial effects, two of the aforementioned designed analogues, GC‐1 and KB2115, commercially known as sobetirome and eprotirome, respectively, entered human clinical trials for dyslipidaemia, displaying encouraging results in the absence of harmful effects typically associated with thyroid hormones (THs) high levels (for reviews, see 19, 20). Unfortunately, no phase II trials for GC‐1 were planned and a phase III trial with KB2115 was terminated due to unexpected side effects not observed in preclinical studies on animals.^21^ In spite of this disappointing result, a 12‐week study with MGL‐3196, formerly known as Resmetirom, has recently been shown to potently reduce hepatic fat content after 12 and 36 weeks of treatment in patients with non‐alcoholic steatohepatitis (NASH), a subtype of non‐alcoholic fatty liver disease (NAFLD), in the presence of only mild adverse effects.^22^

Overall, although caution about the use of these analogues should be maintained as they could be endowed with some deleterious effects, still the possibility of their therapeutic use in different human pathologies, including conditions associated with the impaired regenerative capacity of the liver or to NASH, the second indicator for liver transplantation in the United States, has to be considered.

Novel halogen‐free TRβ selective agonist IS25 and its prodrug TG68 (Figure 1) have been recently identified.^23^, ^24^ Notably, both compounds initially exposed to in vitro analysis of cytotoxicity and ADME‐Tox/off‐target liability revealed a convincing lack of toxicity, supporting their progression in the drug discovery process.

Therefore, we investigated here the effect of a sub‐chronic treatment with these two novel TRβ agonists on rat liver cell proliferation. We found that these agents induced hepatocyte proliferation in the absence of any sign of liver cell damage/toxicity, suggesting that they could be clinically useful in conditions where a rapid liver cell proliferation is required or when the regenerative capacity of this organ is impaired.

## 2 MATERIALS AND METHODS

### 2.1 Animals and treatments

**Figure 2:**
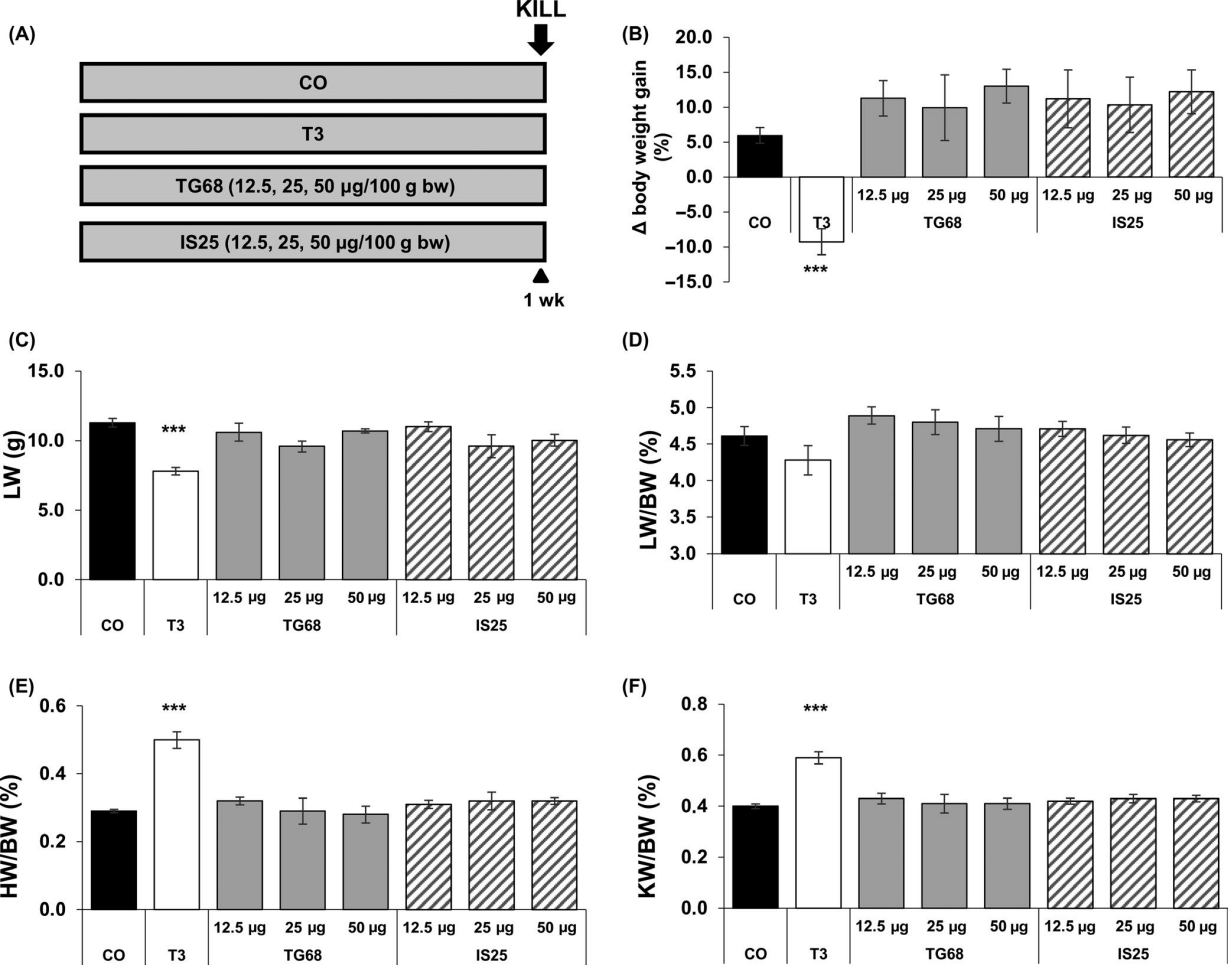
Effect of TG68, IS25 and T3 on body weight, liver weight, heart weight and kidney weight. A, Experimental protocol: F344 male rats were given TG68 and IS25 (12.5, 25 and 50 μg/100g b.w., dissolved in drinking water) or T3 (4 mg/kg diet) or fed a basal diet for one week; B) body weight gain, C) liver weight, D) liver to body weight ratio, E) heart to body weight ratio, F) kidney to body weight ratio. Results were expressed as means ± SD of 3‐5 rats per group. (one‐way ANOVA). Statistically significant for ***P < .001

Five‐week‐old male F‐344 rats (N = 40) purchased from Charles River (Milano, Italy) were maintained on a standard laboratory diet (Ditta Mucedola, Milano, Italy). The animals were given food and water ad libitum with a 12 hours light/dark daily cycle and were acclimated for 1 week before the start of the experiment. All procedures were performed in accordance with the Guidelines for the Care and Use of Laboratory Animals and were approved by Italian Ministry of Health (authorization number: 560/2019‐PR). TG68 and IS25 were dissolved in drinking water at 3 different concentrations (12.5, 25 and 50 µg/100g b.w.). The highest dose of both the compounds was selected on the basis of the mitogenic activity on rat and mouse liver showed by a similar dose of the analogue GC‐1,^25^, ^26^ whereas the oral administration was chosen as the ideal route of administration for future translational studies. Controls received only drinking water. A further group of animals was fed a T3‐supplemented diet (4mg/kg of diet) (For the Experimental Protocol see Figure 2A). For the measurement of hepatocyte proliferation, animals received bromodeoxyuridine (BrdU, 1g/L) in drinking water during the one‐week treatment period. In studies addressed to investigate pancreatic acinar cell proliferation, animals were given daily intragastric doses of the TRβ agonist GC‐1 (50 µg/100g b.w.) for 1 week.

To determine the expression of TRβ target genes, rats were given a single dose of T3 (20 µg/100g b.w.) or TG68 and IS25 (50 µg/100g b.w.) and sacrificed 6 hours after treatment.

Immediately after sacrifice, sections of the liver were fixed in 10% buffered formalin and processed for staining with haematoxylin‐eosin or immunohistochemistry. The remaining liver was snap‐frozen in liquid nitrogen and kept at −80°C until use.

### 2.2 Histology and Immunohistochemistry

Liver, pancreas, kidney and heart sections were fixed in 10% of buffered formalin and processed for staining with haematoxylin‐eosin (H&E) or immunohistochemistry (IHC). For BrdU detection, paraffin‐embedded 4 µm sections were deparaffinized, treated with HCl 2N for 1h and then with 0,1% trypsin at 37°C. Sections were sequentially incubated with goat serum (Abcam), mouse monoclonal anti‐BrdU antibody (Becton Dickinson, San Jose, CA) and with Dako EnVision+® System Labelled Polymer‐HRP anti‐mouse (Dako Corporation, Carpinteria, CA). Peroxidase binding sites were detected by Vector Novared Peroxidase (HRP) Substrate Kit (Vector Labs, Burlingame, CA). Harris haematoxylin solution (Sigma) was used to counterstain liver sections. Labelling index (LI) was expressed as the number of BrdU‐positive hepatocyte nuclei/100 nuclei. Mitotic index was calculated as the number of mitotic figures/1000 nuclei. Results were expressed as the means ± SD of 3 to 5 animals per group. At least 3000 hepatocyte nuclei per liver were scored.

### 2.3 Determination of apoptosis

The incidence of apoptotic bodies was quantified on H&E stained sections or on liver sections immunostained with anti‐caspase‐3 antibody. H&E stained apoptotic bodies were scored according to the following morphological criteria: cytoplasmic eosinophilia and fragmentation of nuclei. Only membrane‐surrounded apoptotic bodies containing nuclear fragments were recorded. The Apoptotic index (AI) was calculated as number of apoptotic bodies/field at X40 magnification. At least 20 fields per rat liver were scored. Apoptosis was also determined by IHC analysis of the cleaved form of caspase‐3 (Cas‐3) as described by Eckle et al,^27^ using rabbit monoclonal anti‐caspase‐3 antibody (Cell Signaling, Danvers, MA). AI was calculated as number of Cas‐3‐positive cells/field at X40 magnification. At least 20 fields per rat liver were scored.

### 2.4 RNA extraction and quantitative PCR analysis

Total RNA was extracted from 50 to 70 mg of frozen liver from rats treated with 50 μg of TG68 or IS25 or vehicle or T3 with TRIzol reagent (Gibco Thermo Scientific, Gaithersburg, MD) following recommended procedures and quantified with NanoDrop ND1000 (Thermo Scientific). One μg of RNA was reverse transcribed using High‐Capacity cDNA Reverse Transcription Kit (Life Technologies, Carlsbad, CA). The expression of *Dio1* and *Spot14* was assessed by real‐time PCR analysis of 10ng of cDNA mixed with 2x TaqMan Gene expression Master Mix and 20X specific TaqMan gene expression assays (*Dio1* Rn00572183_m1, *Spot14* Rn01511034_m1*)* with an ABI PRISM 7300 Thermocycler (Life Technologies). Each sample was run in triplicate, and gene expression analysis of b‐actin was used as endogenous control. Relative quantification analysis for each gene was calculated by 2^‐ΔΔCt^ method.

### 2.5 Western blot analysis

Total cell extracts were prepared from frozen livers. Liver tissue was homogenized with Bullet Blender Tissue Homogenizer (Next Advance, Troy, NY, USA) in hot LB buffer (1/2 H20, ¼ SDS 10%, ¼ TRIS‐HCl 0.5M pH6.8) for 2 minutes at maximum speed. Protein amount was quantified with BCA (Pierce, Thermo Fisher, Waltham, MA, USA) according to manufacturer's instructions. 30 μg of whole cell lysate was loaded in a 8% gel and incubated with the following antibodies: Cyclin D1: sc‐8396; PCNA: sc‐56; Cyclin A1: sc15383 (Santa Cruz Biotechnology, Dallas, TX, USA): B‐actin: A3854 (Sigma, Darmstadt, Germany). Final detection was done with the ECL system (Amersham, Uppsala, Sweden).

**Analysis of serum free triiodothyronine (FT3), free tiroxine (FT4), triglycerides (TGs), cholesterol (CH), glucose, aspartate aminotransferase (AST), alanine aminotransferase (ALT), alkaline phosphatase (ALP), gamma‐glutamyl transferase (GGT) and total serum bilirubin (TSB): **Immediately after sacrifice, blood samples were collected from the abdominal aorta, serum was separated by centrifugation (2000 *g* for 20 minutes) and tested for triglycerides, cholesterol, aspartate aminotransferase and alanine aminotransferase using a commercially available kit from Boehringer (Mannheim, Germany).

### 2.6 Statistical Analysis

All data were expressed as the mean ± SD unless otherwise indicated. Differences between groups were compared using one‐way ANOVA.

## 3 RESULTS

### 3.1 Sub‐chronic treatment with TG68 and IS25 does not elicit T3‐associated changes in liver, heart and kidney weight

Initial studies were performed to explore the effect of different doses of TG68 and IS25 on liver cell proliferation. To this aim, rats were exposed to an experimental protocol consisting of the administration of both the analogues (50, 25 and 12.5 μg/100g b.w.) in drinking water for 1 week. Two other groups were fed either a T3‐supplemented diet or a basal diet (Figure 2A). At sacrifice, administration of T3 resulted in a decreased body weight gain compared to controls, whereas no such effect was observed at any of the doses of IS25 or TG68 (Figure 2B). In addition, a decreased liver weight was observed following T3 treatment (Figure 2C). As a result, liver to body weight ratio was decreased in T3 but not in animals treated with the two thyromimetics (Figure 2D).

T3 administration also caused a strong increase in heart weight, and, as a consequence, an enhanced heart/body weight ratio (Figure 2E). Indeed, necroscopy on T3‐treated rats showed a marked hypertrophy of the left ventricle and the septum with increased muscle stiffness and reduction in the left ventricular cavity size. No such effect was found in the heart of animals treated at all doses of both analogues, showing that the preferential binding to—and activation of—TRβ does not result in cardiac hypertrophy.

Another organ severely affected by T3 is the kidney.^28^ Accordingly, the kidney weight and the ratio of kidney/body weight were greatly and significantly increased after the administration of thyroid hormone (Figure 2F). No such effect was observed following TG68 or IS25.

**Figure 3:**
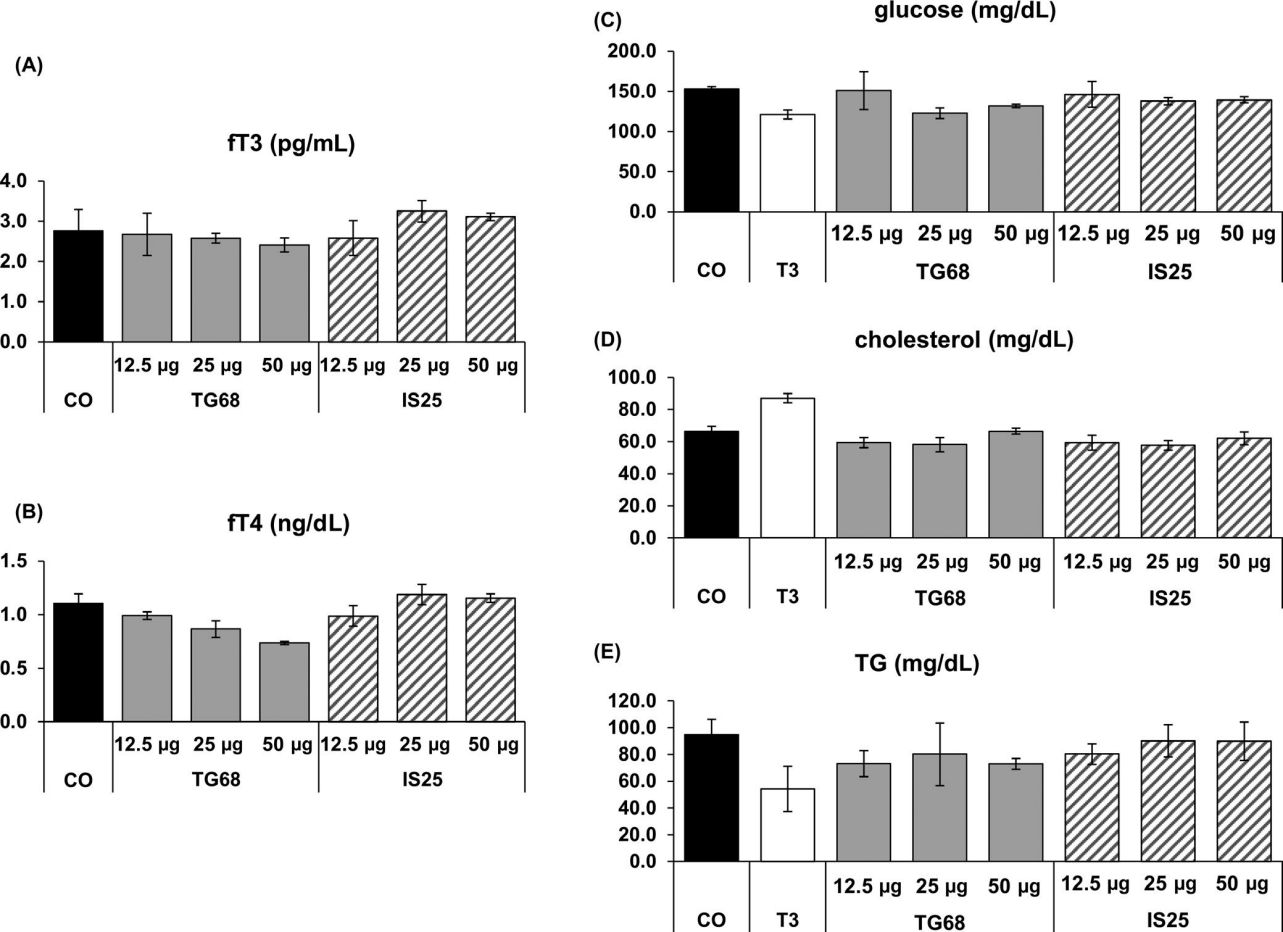
Serum levels of free T3 (fT3), free T4 (fT4), glucose, cholesterol and triglycerides (TGs). Rats were treated according to Figure 2A. Results were expressed as means ± SD of 3‐5 rats per group. (one‐way ANOVA)

No significant effect on the serum levels of free T3 (fT3) or T4 (fT4) as well as on those of glucose, cholesterol and triglycerides (TG) was observed following treatment with T3 or the two novel drugs (Figure 3).

### 3.2 TG68 and IS25 induce hepatocyte proliferation

**Figure 4:**
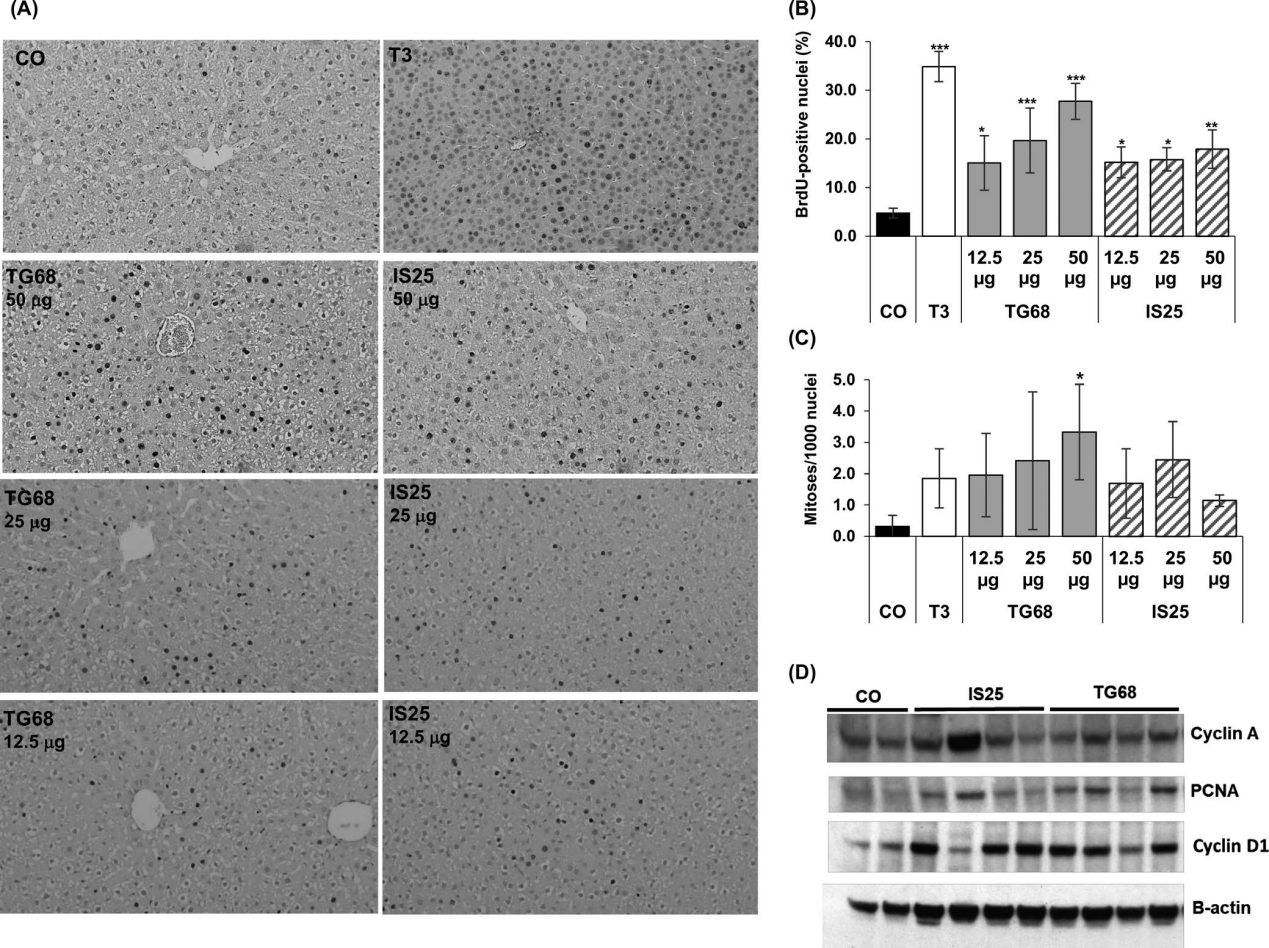
Effect of sub‐chronic treatment with TG68 and IS25 on hepatocyte proliferation. A) Microphotograph illustrating the presence of several BrdU‐positive hepatocyte nuclei (X20, counterstained with haematoxylin); rats were sacrificed 1 week after continuous treatment with TG68, IS25 and T3. TG68 and IS25 were administered at the dose of 12.5, 25 and 50 μg/100g b.w. dissolved in drinking water. T3 was administered in the diet at a final concentration of 4mg/kg diet. All animals received BrdU (1 g/L) in drinking water all throughout the treatment period; B) Labelling index (LI). LI was expressed as number of BrdU‐positive hepatocyte nuclei/100 nuclei; C) Mitotic index (MI). MI was expressed as number of mitoses/1000 nuclei. At least 3000 hepatocyte nuclei per liver were scored. Results were expressed as means ± SD of 3‐5 rats per group. (one‐way ANOVA). Statistically significant for *P < .05; **P < .01; ***P < .001; D) Western blot analysis showing increased expression of cyclin A, PCNA and cyclin D1 in the liver of rats treated with TG68 and IS25 (50 μg/100g b.w., dissolved in drinking water) for 7 days. For Western blot, total cell extracts were prepared from frozen livers, and the membrane was probed with the indicated antibodies. Actin was used as loading control. Each lane represents an individual sample. CO, controls

Our previous findings showed that T3 and two other analogues—Sobetirome and Eprotirome—exert a powerful mitogenic effect in the liver, suggesting their possible use in regenerative medicine.^29^, ^30^ Therefore, we investigated whether TG68 and IS25 could elicit a proliferative response in this organ as well. As shown in Figure 4A,B, one‐week treatment with either TG68 and IS25 at all doses induced a strong hepatocyte proliferation, as assessed by BrdU incorporation. Indeed, Labelling index (LI) displayed a 3‐ to 6‐fold increase in DNA synthesis compared to controls. Notably, the extent of hepatocyte proliferation elicited by the highest dose of TG68 was significantly higher than that of the same dose of IS25, and almost similar to that observed with T3 (Figure 4B). A dose‐response in the mitogenic effect was observed for TG68 but not for IS25. A highly significant mitogenic effect was exerted by both thyromimetics even when administered at the lowest dose (12.5 μg/100 g b.w). The increased LI was accompanied by a higher number of cells undergoing mitosis, particularly evident at the dose of 50 μg/100 g b.w. of TG68 (Figure 4C).

The mitogenic effect of TG68 and IS25 was further confirmed by Western blot analyses of cell cycle‐related proteins. Indeed, as shown in Figure 4D, the protein content of cyclin D1, cyclin A and PCNA was enhanced in the liver of rats treated with the two TRβ agonists compared to that of control animals.

Histological examination showed features very similar to those of normal liver without those traits typical of T3, such as an almost complete depletion of cytoplasmic components in the hepatocytes due to the catabolic effect of the thyroid hormone and resulting in increased nuclear/cytoplasmic ratio (Figure 4A). Notably, while cellular changes similar to those induced by T3 were also observed after Eprotirome treatment,^30^ no such changes were observed even at the highest dose of the two novel thyromimetics.

### 3.3 Thyromimetic‐induced hepatocyte proliferation is not associated with liver injury

**Figure 5:**
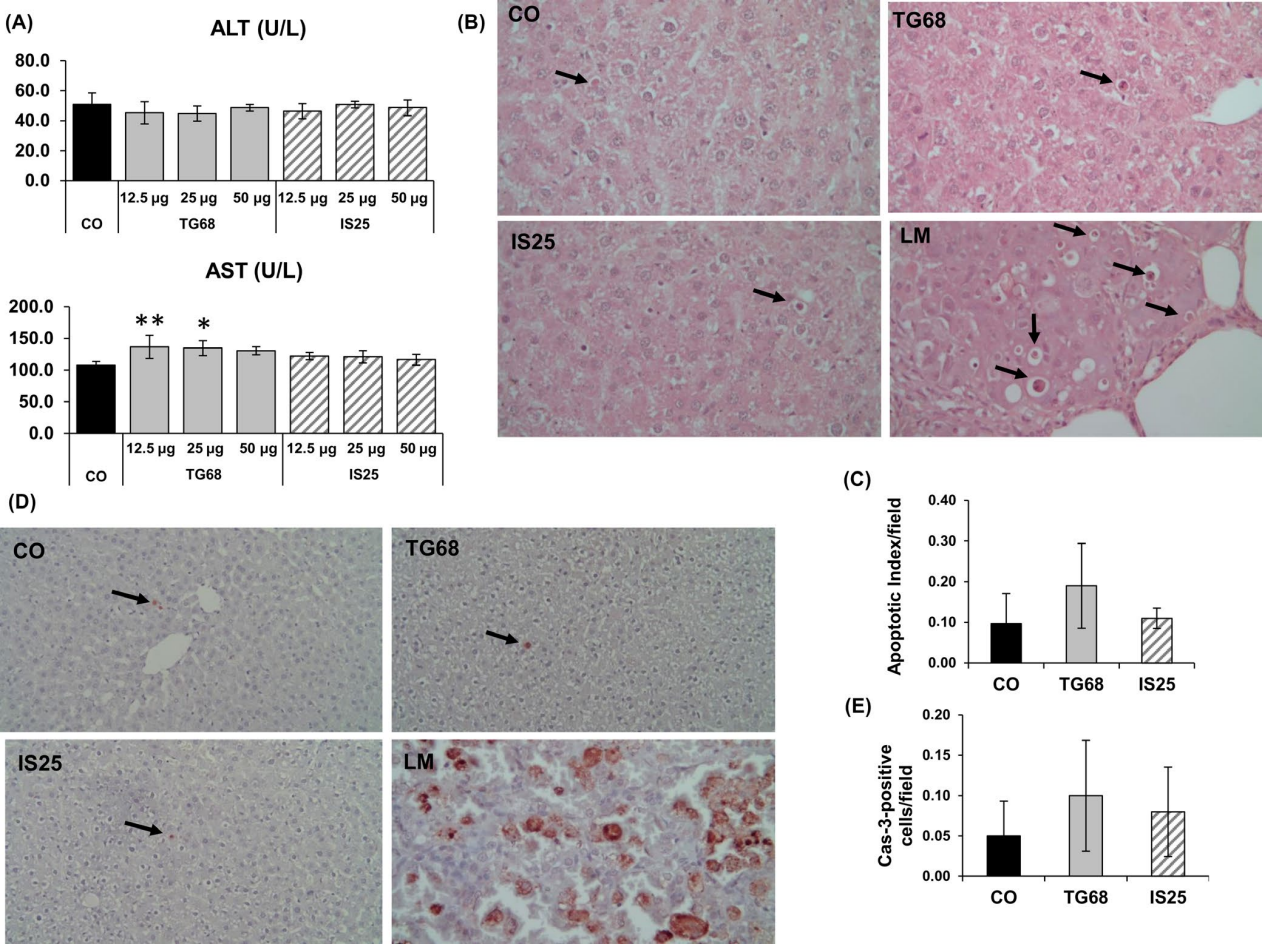
Hepatocyte proliferation induced by TG68 and IS25 is not associated with liver injury. A) Graphs representing the levels of serum transaminases 7 days after treatment of rats with TG68, IS25 and T3, as described in Figure 2A. AST and ALT were determined in blood samples collected from the abdominal aorta (one‐way ANOVA) statistically significant for *P < .05; **P < .01; B) representative microphotograph displaying numerous membrane‐surrounded apoptotic bodies (ABs) containing nuclear fragments in a rat lung metastasis (LM) (H&E, X40); only a negligible number of ABs can be observed in the liver of control rats or rats treated with the two thyromimetics (H&E, X40). ABs are indicated by the arrows; C) Apoptotic index (AI). AI was calculated as the number of ABs per field at X40 magnification. From 20 to 40 fields per rat liver were scored. Results were expressed as means ± SD of 3‐4 rats per group (one way ANOVA); D) representative microphotographs showing immunostaining for the cleaved form of caspase‐3 (Cas‐3) in the liver of rats treated with the vehicle or TG68, IS25 (50 μg/100g b.w, in drinking water) (Cas‐3 counterstained with haematoxylin, X20). Arrows indicate single Cas‐3‐positive cells. Rat lung metastasis (LM) displaying several Cas3‐positive cells is shown as a positive control (X40); E) Apoptotic index (AI). AI was calculated as the number of Cas‐3‐positive cells/per field at X40 magnification. From 20 to 40 fields per rat liver were scored. Results were expressed as means ± SD of 3‐4 rats per group. (one‐way ANOVA)

To investigate whether hepatocyte proliferation induced by TG68 and IS25 could be a compensatory response to liver cell necrosis, we assessed the levels of serum transaminases. The results showed no significant changes of ALT and only a very slight increase of AST in rats treated with TG68 (Figure 5A). We have also determined serum levels of total bilirubin (TSB), gamma‐glutamyltransferase (GGT) and alkaline phosphatase (ALP) (Figure S1). While no change in GGT and TSB was observed in animals exposed to either T3 or thyromimetics, ALP levels were strongly enhanced by T3 (almost 4‐fold increase), with only small modifications in TG68‐treated animals.

Next, we investigated whether the two agonists could induce apoptosis. Apoptotic bodies (ABs) can be clearly identified as membrane‐surrounded globules containing nuclear fragments (Figure 5B, bottom right). Therefore, we scored their incidence on H&E liver sections of control rats and of animals treated with TG68 and IS25. As shown in Figure 5B, only a negligible number of ABs was detected in control liver as well as in the liver of rats treated with the two thyromimetics. Accordingly, the Apoptotic index (AI) revealed no change in the number of ABs between treated and untreated animals (Figure 5C).

Apoptosis was also investigated by immunostaining for the cleaved form of Cas‐3. Similar to what was observed in H&E stained sections, a very low number of Cas‐3‐positive cells was observed in the liver of rats treated with TG68 or IS25 (Figure 5D). Accordingly, AI did not reveal any significant difference between control and treated rats (Figure 5E).

Since the dose of 50 μg of TG68 and IS25 displayed the maximal mitogenic potency in the absence of liver injury, this concentration was selected for further experiments.

### 3.4 TG68 and IS25 induce the expression of TR target genes

**Figure 6:**
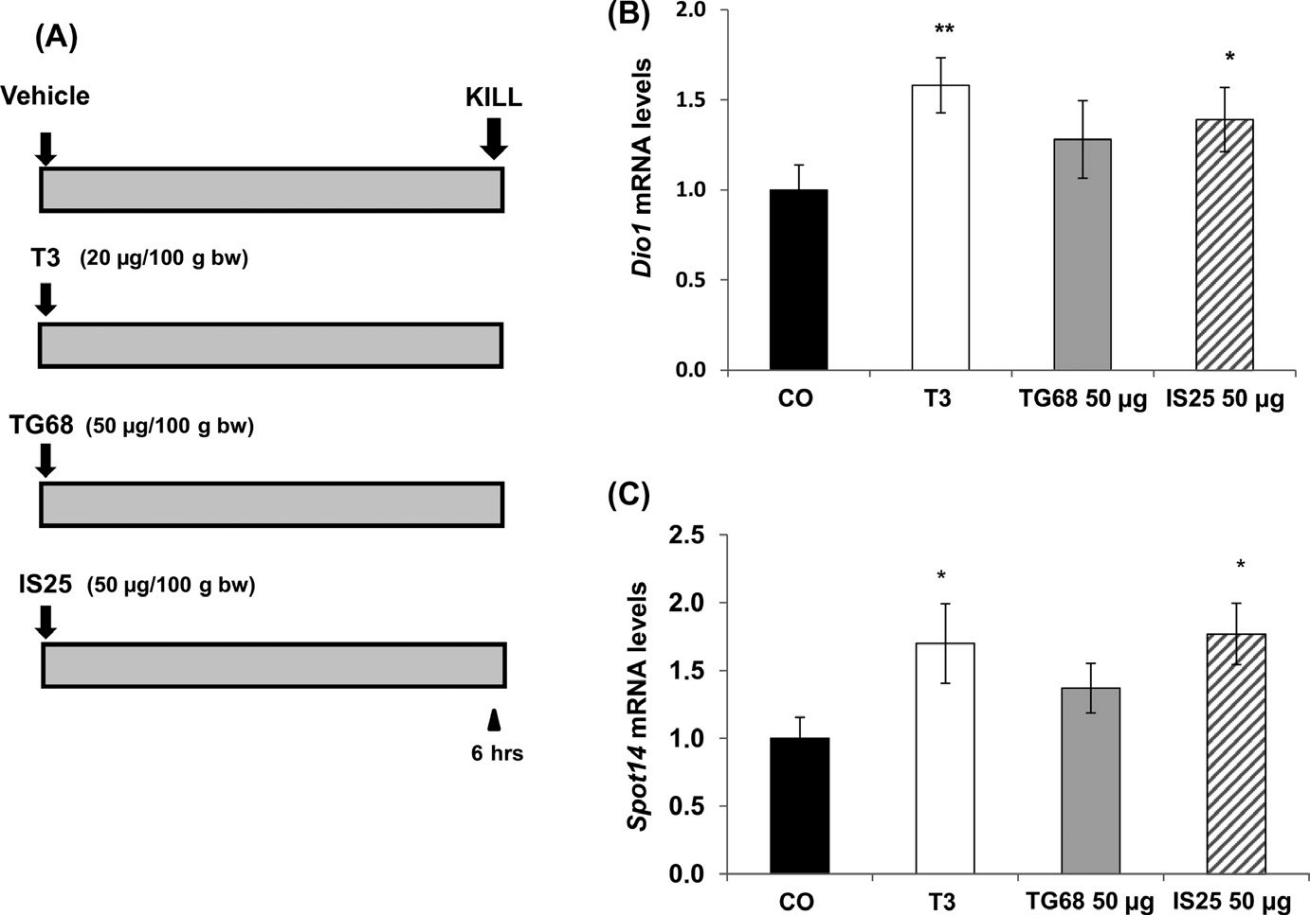
Induction of TRβ target genes by TG68 and IS25. A, Experimental protocol: F344 male rats were given a single i.g. administration of TG68 and IS25 (50 μg/100g b.w dissolved in a solution of DMSO/corn oil) or T3 (20 μg/100g b.w, dissolved in 0.001N NaOH/corn oil), or an equal volume of the vehicle. Rats were killed six hours thereafter; B,C) qRT‐PCR analysis of the expression of *Dio1* and *Spot14*. Each sample was run in triplicate, and gene expression analysis of B‐actin was used as endogenous control. Relative quantification analysis for each gene was calculated by 2^‐ΔΔCt^ method. (one‐way ANOVA). *P < .05; **P < .01

It was previously shown that IS25 binds and specifically activates TRβ receptor in vitro.^23^, ^24^ To assess whether in vivo hepatocyte proliferation induced by the novel TRβ agonists is preceded by TR activation, we evaluated the expression of two well‐established TRβ‐target genes, deiodinase 1 (*Dio1*) and Spot14 (*Spot14*) following treatment with a single intragastric (i.g.) dose of 50 μg/100 g b.w. of either TG68 or IS25. An equal volume of the vehicle and a single i.g. dose of T3 (20 μg/100 g b. w.) were given as a negative and positive control, respectively (Figure 6A). As shown in Figure 6B,C qRT‐PCR analysis revealed a statistically significant induction of these two genes 6 hours after either T3 or IS25. Increased expression of Dio1 and Spot14 was observed also after TG68 treatment, although it did not reach statistical significance.

### 3.5 TG68 and IS25 do not induce pancreatic acinar cell proliferation

**Figure 7:**
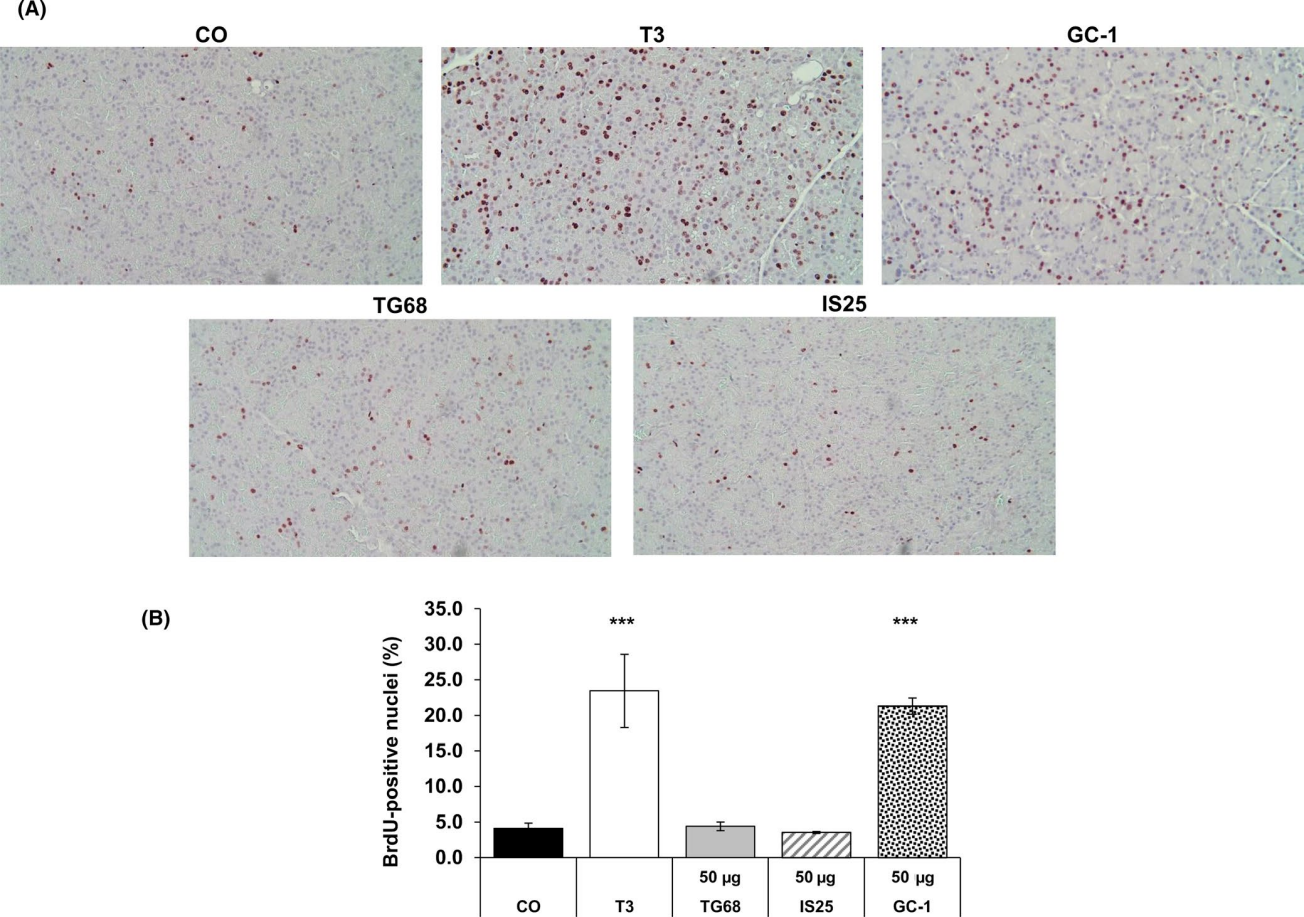
TG68 or IS25 do not elicit pancreatic acinar cell proliferation. A, Representative microphotographs of immunohistochemical staining for BrdU in the pancreas of untreated rats or rats treated with T3, GC‐1, TG68 or IS25 for one week. T3 was administered in the diet (4 mg/kg diet); GC‐1 (50 μg/kg) was given by gavage once daily; TG68 and IS25 were dissolved in drinking water at a dose of 50 μg/kg. BrdU (1 mg/ml) in drinking water was given all throughout the experimental time. Several BrdU‐positive acinar cells are observed in the pancreas of T3‐ and GC1‐treated rats, but not in TG68‐ and IS25‐treated animals (X20, sections counterstained with haematoxylin); B, LI of rat pancreatic acinar cells. LI was expressed as number of BrdU‐positive acinar cell nuclei/100 nuclei. At least 2000 acinar cells per pancreas were scored. Results are expressed as means ± SD of 3 to 5 rats per group. (one‐way ANOVA). ***Statistically significant from control; *P* < .001

It has been reported that T3 and GC‐1 exert a strong mitogenic effect on pancreatic acinar cells.^31^, ^32^ Therefore, we investigated whether IS25 and TG68 could also exert a similar mitogenic effect on this cell population. Figure 7A,B shows that while T3 and GC‐1 induced a striking increase in acinar cell proliferation, no mitogenic effect was evident in the pancreas of rats treated with the two novel thyromimetics. These results suggest that in addition to the selective affinity for TRβ, IS25 and TG68 can also be considered as hepato‐specific.

### 3.6 TG68 and IS25, unlike T3, do not induce renal or cardiac hypertrophy or proliferation

T3‐induced renal and cardiac hypertrophy is associated with enhanced proliferation of renal and cardiac cells, including myocardiocytes.^33^, ^34^, ^35^ To investigate whether the two agonists share the mitogenic effect of T3 in these tissues, we determined the proliferative activity by BrdU immunostaining. As shown in Figure S2A,B, while T3 induced a strong proliferation both in the kidney and in the heart, no difference between untreated animals and rats given TG68 and IS25 could be observed. Moreover, while a severe degree of hypertrophy was seen in H&E sections of the kidney and the heart of rats treated with T3, no appreciable difference was found in the two organs of rats treated with the two thyromimetics when compared to untreated animals (Figure S3A, B).

## 4 DISCUSSION

The present study shows that the recently discovered TRβ agonists IS25 and TG68 exert a profound mitogenic effect in rat liver, in the absence of one of the most T3‐harmful side effects, namely cardiac hypertrophy.

Hepatocyte proliferation driven by IS25 and TG68 was associated with activation of TR target genes, such as Dio1 and Spot14, suggesting that the mitogenic effect of these drugs may be due to binding and activation of TRβ.

Importantly, the liver proliferative response induced by the two TRβ agonists was not associated with liver damage, as assessed by biochemical determination of serum transaminases and by immunostaining for caspase‐3, but it was the result of a direct effect of these drugs enabling quiescent hepatocytes to re‐entry into the cell cycle.

In the last few years, many thyromimetics have been designed to be used to reduce hypercholesterolaemia and/or hypertriglyceridemia in the absence of T3‐induced adverse thyrotoxic effects on the heart, skeletal muscle, bone or pituitary gland.^13^, ^19^, ^20^, ^32^, ^36^, ^37^ After initial encouraging results, the development of some of these drugs (ie KB2115, GC‐1) was hampered due to unwanted side effects.^21^

Another powerful effect exerted by synthetic TRβ agonists is their mitogenic ability. Indeed, similar to T3, both GC‐1 and KB2115 induce rat hepatocyte proliferation.^25^, ^30^

Our present data demonstrating a strong hepatomitogenic effect of IS25 and TG68 suggest that these compounds could be useful in regenerative medicine to manage conditions whereby a rapid liver regeneration is required, such as in the living‐related transplantation, or in the elderly status that is characterized by an impaired liver regenerative capacity.^38^, ^39^ Indeed, previous studies have shown that T3 and GC‐1 improved the regenerative response of rodent livers after 70% or 90% hepatectomy and stimulated liver cell proliferation in aged rats when given prior to—or after 70% PH.^29^, ^40^, ^41^, ^42^

Of note, unlike T3 or GC‐1, the two novel TRβ agonists investigated were mitogenic only for the liver and not for other tissues, such as the acinar cell compartment of the pancreas. This finding is intriguing, as from a structural point of view, the 3 compounds display the same scaffold. A possible explanation that can be envisaged is that the chemical structure of the prodrug TG68 and of its metabolite IS25 influences their ability to reach the pancreas, since most of them are captured by the liver. Indeed, the chemical group inserted in the molecules is typically handled by the hepatocytes due to their highly efficient drug‐metabolizing system. Since aniline derivatives undergo hepatic biotransformation, it is possible that most of the effects exerted by IS25 and TG68 are hepato‐specific instead of systemic as observed with GC‐1. Future investigations are needed to gain a detailed understanding of the biotransformation pathways involving IS25 and TG68.

Irrespectively of the mechanisms responsible for such differences, the powerful hepatocyte mitogenic capacity in the absence of T3‐associated harmful side effects, associated with the absence of proliferative response in extrahepatic organs/tissues (pancreas, kidney, heart), represent an important improvement in the field of regenerative therapies.

Another relevant point raised by these results is the potential usefulness of these analogues to reduce fatty liver and consequently NASH. Indeed, our previous results have shown that IS25 and its prodrug TG68 exert a strong effect on lipolysis in human hepatoma (HepG2) cells,^23^, ^24^ in agreement with the effect of GC‐1 and other thyromimetics on NAFLD/NASH.^16^, ^17^, ^18^, ^22^

A further possible application that deserves to be explored in future studies is the use of these novel TRβ agonists as therapeutic anti‐cancer drugs. In fact, it has been demonstrated that a) a short term treatment with GC‐1 of rats carrying preneoplastic nodules induced a rapid disappearance of most lesions, concomitantly with a rapid disappearance of fat accumulation,^43^ b) GC‐1 also inhibits the progression of Met‐β‐catenin‐driven HCCs in mice^44^ and c) treatment with KAT‐68, another liver‐selective thyromimetic with hypocholesterolemic properties, showed inhibitory effects in the early and late phases of hepatocarcinogenesis.^45^

In conclusion, although caution about the use of these analogues should be maintained, as they could have some yet unknown deleterious effects, still the possibility of their therapeutic use in regenerative medicine and in HCC, a disease that currently does not offer any satisfactory alternative, might be carefully considered.

## CONFLICT OF INTEREST

The authors declare no conflict of interest.

## AUTHORS’ CONTRIBUTION

AP, MAK, and LC: performed in vivo experiments, histopathologic and classification, histochemical staining, qRT.PCR analysis, and contributed to the study design; CM: performed WB analysis; SG: contributed to the writing of the manuscript; MR, SS, GC, and SR: synthesized the two thyromimetics; AC and SR: conceived and supervised the study, provided funding, and wrote the manuscript.

## Supporting information

FigS1‐S3

## Data Availability

The data that support the findings of this study are available from the corresponding author upon reasonable request.
