# Corrigendum

**DOI:** 10.1111/cpr.13006

**Published:** 2021-03-09

*Cell Prolif*., 2020, **53**, e12808

**Potential role of two novel agonists of thyroid hormone receptor‐β on liver regeneration**

Andrea Perra1^1^, Marta Anna Kowalik^1^, Lavinia Cabras^1^, Massimiliano Runfola^2^, Simona Sestito^3^, Cristina Migliore^4,5^, Silvia Giordano^4,5^, Grazia Chiellini^3^, Simona Rapposelli^2^, Amedeo Columbano^1^

^1^Department of Biomedical Sciences, Unit of Oncology and Molecular Pathology, University of Cagliari, Cagliari, Italy

^2^Department of Pharmacy, University of Pisa, Pisa, Italy

^3^Department of Pathology, University of Pisa, Pisa, Italy

^4^Department of Oncology, University of Turin, Turin, Italy

^5^Candiolo Cancer Institute, FPO‐IRCCS, Candiolo, Italy

The authors would like to draw the reader's attention to the below errors:

Affiliation 5 was erroneously written in the published version of the article. The correct affiliation is ^‘^Candiolo Cancer Institute, FPO‐IRCCS, Candiolo, Italy’, which was already corrected in the above list of affiliations.

The authors would like to apologize for any inconvenience caused.

