# Supplementary material for: Potential role of two novel agonists of thyroid hormone receptor‐β on liver regeneration
Source: Cell Prolif. 2020 Apr 29;53(5):e12808. doi: 10.1111/cpr.12808 (PMC7260063; doi:10.1111/cpr.12808)
Supplement: Supplementary file 1 — FigS1‐S3 [file CPR-53-e12808-s001.pptx]

## Slide 1
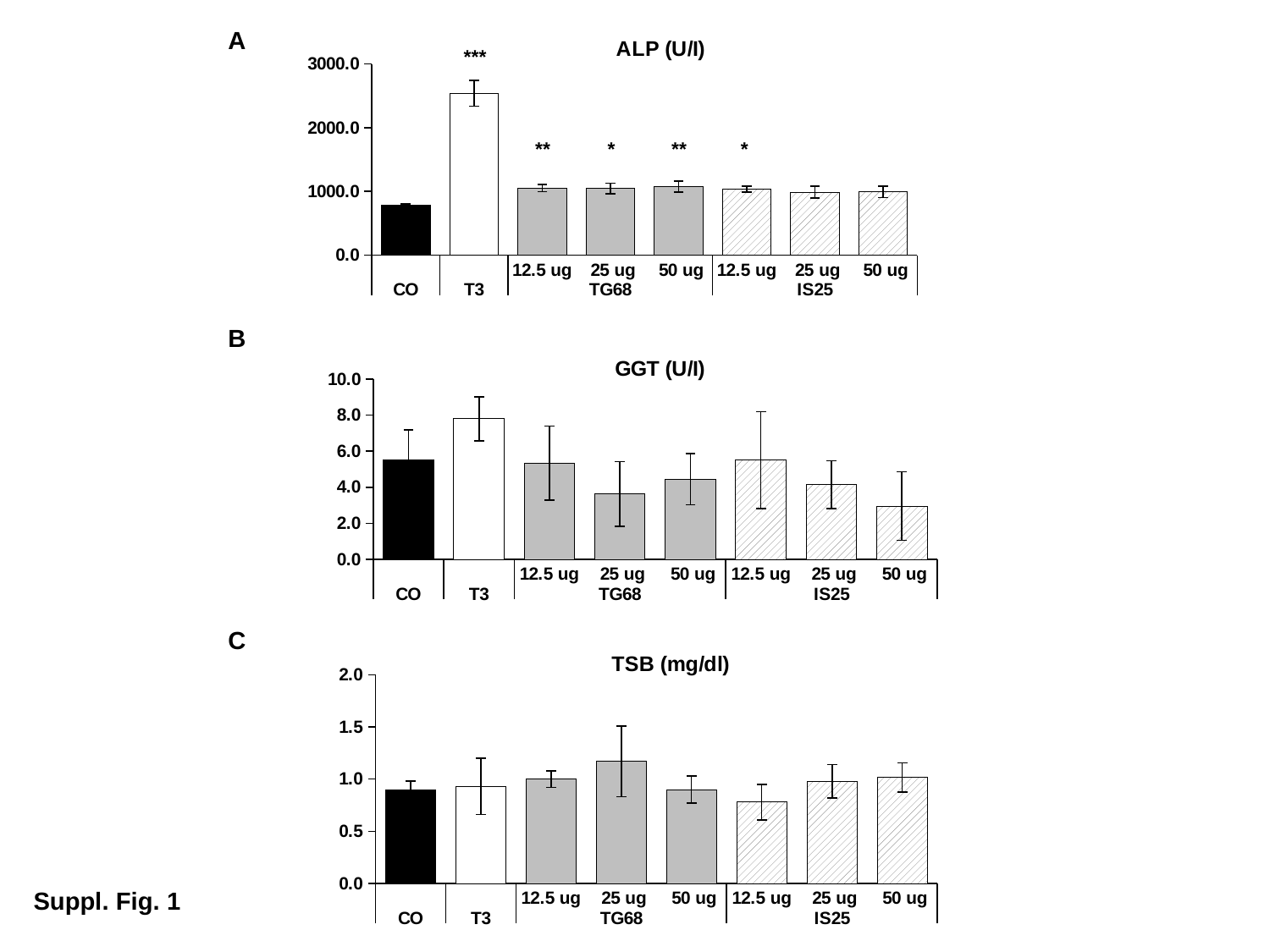

### Chart: ALP (U/I)
| Category | |
|---|---|
| | 780.0 |
| | 2542.75 |
| 12.5 ug | 1051.4 |
| 25 ug | 1048.25 |
| 50 ug | 1075.5 |
| 12.5 ug | 1034.8 |
| 25 ug | 990.0 |
| 50 ug | 992.5 |***
**
*
**
*
A
### Chart: GGT (U/I)
| Category | |
|---|---|
| | 5.5 |
| | 7.8 |
| 12.5 ug | 5.34 |
| 25 ug | 3.63 |
| 50 ug | 4.45 |
| 12.5 ug | 5.5 |
| 25 ug | 4.14 |
| 50 ug | 2.95 |B
### Chart: TSB (mg/dl)
| Category | |
|---|---|
| | 0.9 |
| | 0.93 |
| 12.5 ug | 1.0 |
| 25 ug | 1.17 |
| 50 ug | 0.9 |
| 12.5 ug | 0.78 |
| 25 ug | 0.98 |
| 50 ug | 1.015 |C
Suppl. Fig. 1

## Slide 2
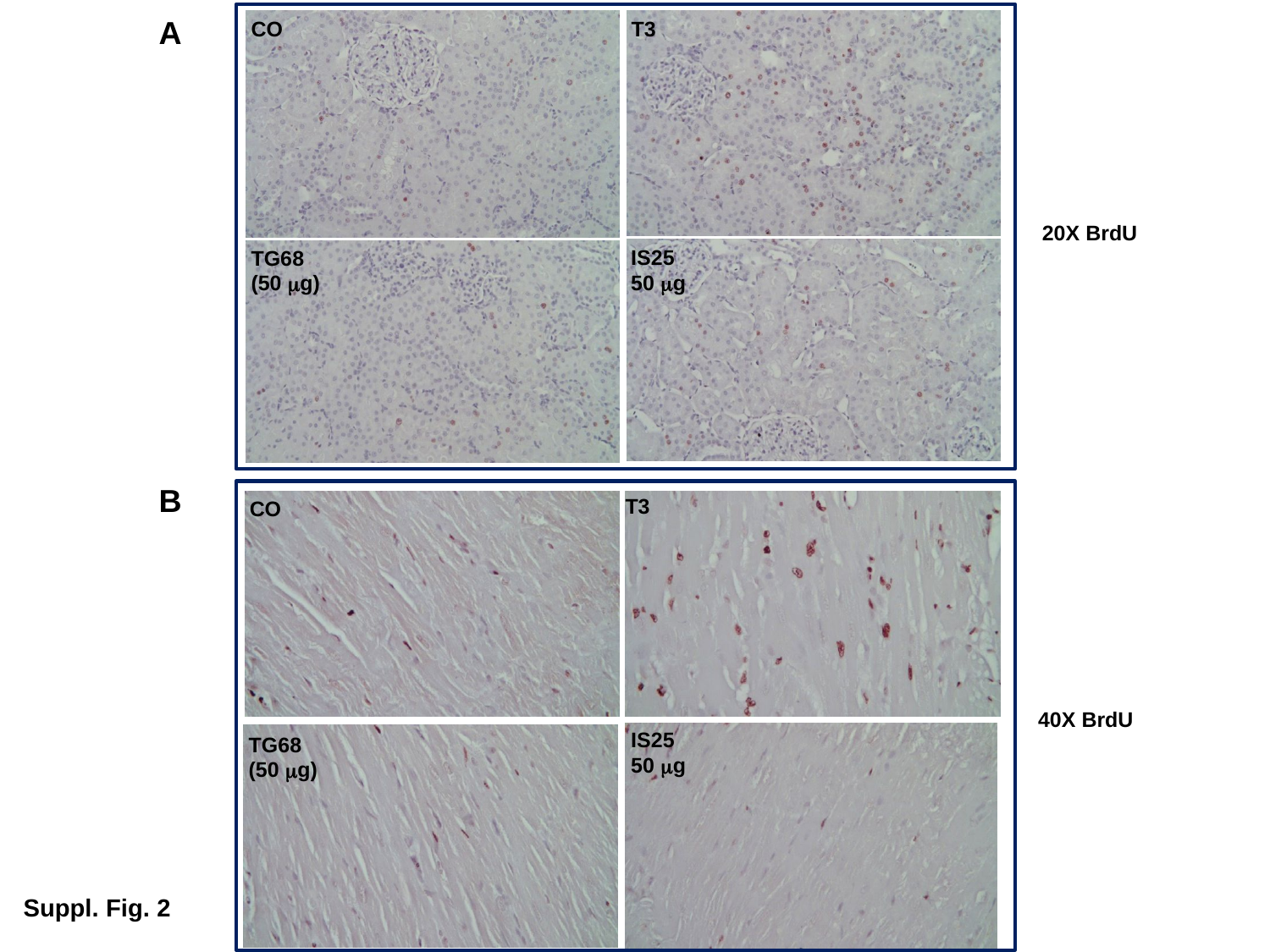

A
CO
T3
20X BrdU
IS25
50 mg
TG68
(50 mg)
B
T3
CO
40X BrdU
IS25
50 mg
TG68
(50 mg)
Suppl. Fig. 2

## Slide 3
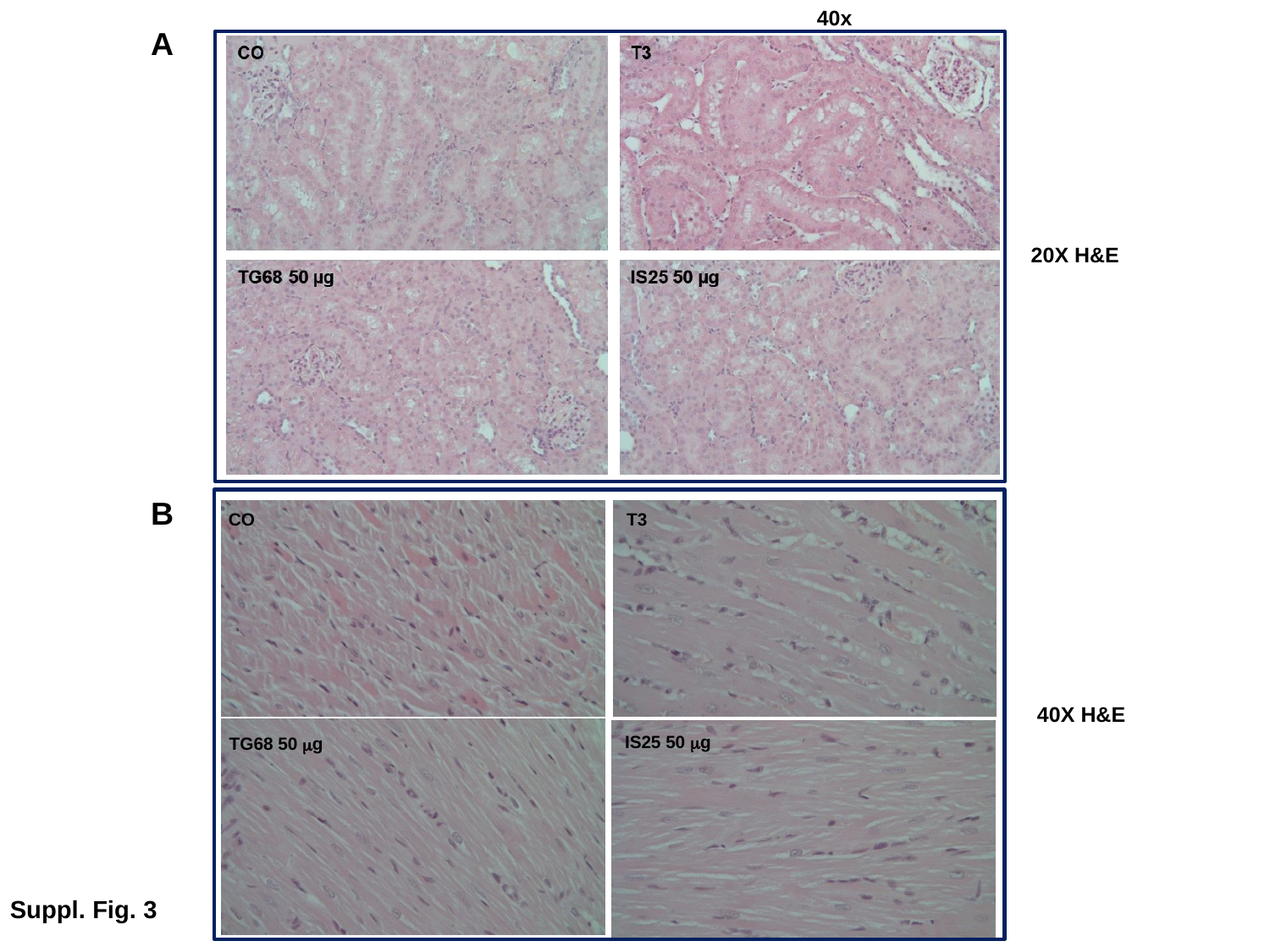

40x
A
20X H&E
B
CO
T3
40X H&E
IS25 50 mg
TG68 50 mg
Suppl. Fig. 3
