# Supplementary material for: Potential role of two novel agonists of thyroid hormone receptor‐β on liver regeneration
Source: Cell Prolif. 2020 Apr 29;53(5):e12808. doi: 10.1111/cpr.12808 (PMC7260063; doi:10.1111/cpr.12808)
Supplement: Supplementary file 2 [file CPR-53-e12808-s002.docx]

**SUPPLEMENTARY MATERIAL**

**FIGURE LEGENDS**

**SUPPLEMENTARY FIG. 1. Serum levels of GGT, ALP and TSB.** F344 male rats were given T3 (4mg/kg diet), TG68 and IS25 (12.5, 25 and 50 μg/100g b.w., dissolved in drinking water) or fed a basal diet (CO) for one week. Results were expressed as means ± SD. of 3–5 rats per group. (one way ANOVA). Significantly different from controls for **P*<0.05; ***P*<0.01; ****P*<0.001.

**SUPPLEMENTARY FIG. 2. A)** Representative microphotographs of immunohistochemical staining for BrdU in the kidney of untreated rats or rats treated with T3, TG68 or IS25 for one week. T3 was administered in the diet (4 mg/kg diet); TG68 and IS25 were dissolved in drinking water at a dose of 50 μg/kg. BrdU (1 mg/ml) in drinking water was given all throughout the experimental time. The photo shows a much higher number of BrdU-positive renal tubular in the kidney of T3-treated rats compared to that of untreated or TG68 and IS25-treated animals (X20, sections counterstained with hematoxylin); **B)** Representative microphotographs of immunohistochemical staining for BrdU in the heart of rats treated as in A. Increased incorporation of BrdU can be seen in heart samples of rats treated with T3 compared with controls or rats treated with TG68 and IS25 (X40, sections counterstained with hematoxylin);

**SUPPLEMENTARY FIG. 3. A)** Representative microphotographs of H&E staining of the kidney of controls (CO) or of rats treated with T3, TG68 or IS25 for one week, as described in Suppl. Fig 2A. T3 administration for 1 week resulted in evident tubular cells hypertrophy, characterized by the expansion of the brush border (proximal convoluted tubule), and an enlarged Bowman space (upper right border). No histological alteration can be seen in the kidneys of controls or of rats treated with TG68 or IS25 (X20, H&E); **B)** Representative microphotographs of H&E staining of the heart of rats untreated (CO) or treated with T3, TG68 or IS25 for one week, as described in Suppl. Fig 2A. Evident cardiomyocyte hypertrophy in the heart of of T3-treated rats can be observed. Thickening of fibers is associated with enlargement of nuclei and increased number of non-parenchymal cells. No obvious alterations are present in the heart of controls or of rats exposed to TG68 and IS25 (X40, H&E).
